# Cardiovascular disease and covid-19: A systematic review

**DOI:** 10.1016/j.ijcha.2024.101482

**Published:** 2024-08-02

**Authors:** B.A. Krishna, M. Metaxaki, N. Sithole, P. Landín, P. Martín, A. Salinas-Botrán

**Affiliations:** a Department of Medicine, University of Cambridge, Cambridge, United Kingdom; b Department of Infectious Diseases, Cambridge University, Cambridge, United Kingdom; c Department of Cardiology, Hospital Clínico San Carlos, Madrid, Spain; d Department of Respiratory Medicine, Hospital Clínico San Carlos, Madrid, Spain; e Department of Infectious Diseases, Hospital Clínico San Carlos, Madrid, Spain; f Department of Medicine, Universidad Complutense de Madrid, Madrid, Spain

**Keywords:** COVID-19, Cardiovascular disease, Long COVID-19 syndrome, SARS-CoV-2

## Abstract

**Background:**

Cardiovascular complications of COVID-19 are numerous and aspects of this phenomenon are not well known. The main objective of this manuscript is a systematic review of the acute and chronic cardiovascular complications secondary to COVID-19.

**Methods:**

A systematic review of the literature through Medline via PubMed was conducted (2020–2024).

**Results:**

There is a plethora of effects of COVID-19 on the heart in the acute setting. Here we discuss pathophysiology, myocardial infarctions, heart failure, Takotsubo Cardiomyopathy, myocardial injury, myocarditis and arrhythmias that are caused by COVID-19. Additionally, these cardiovascular injuries can linger and may be an underlying cause of some Long COVID symptoms.

**Conclusions:**

Cardiovascular complications of COVID-19 are numerous and life-threatening. Long COVID can affect cardiovascular health. Microclotting induced by SARS-CoV-2 infection could be a therapeutic target for some aspects of Long Covid.

## 1 Introduction

The COVID-19 pandemic, caused by the betacoronavirus SARS-CoV-2, has presented unprecedented challenges to global healthcare systems [1], [2]. Beyond its respiratory impact, COVID-19 has revealed a complex interplay between the virus and the cardiovascular system, reminiscent of prior coronavirus outbreaks such as SARS and MERS [3].

Cardiovascular complications in COVID-19 are diverse and can encompass a spectrum of conditions, affecting patients during the acute phase, the aftermath and for month or years after infection [3]. Myocardial injury, arrhythmias, heart failure, vascular dysfunction, and thromboembolic events have emerged as common cardiovascular injuries in COVID-19 patients [3]. Furthermore, the risk of acute myocardial infarction (MI) and ischemic stroke may be elevated among those afflicted with the virus, underscoring the systemic impact of COVID-19 on the cardiovascular system. Arrhythmias, including atrial fibrillation, have been observed, as have cases of heart failure decompensation [3].

These myocardial effects are influenced by pre-existing cardiac conditions, risk factors for heart disease, and the severity of the initial COVID-19 illness [4]. The diagnostic role of cardiac biomarkers, **electrocardiograms** (ECG), and cardiac imaging cannot be understated in managing these complications [5]. One striking observation is the significant contribution of venous thromboembolic disease, including pulmonary thromboembolism, to the severity of COVID-19. These findings emphasize the importance of recognizing and managing cardiovascular complications as integral components of COVID-19 care [6].

Beyond the acute phase, COVID-19 has raised concerns about long-term cardiovascular consequences, with mounting compelling evidence that the severity of the initial COVID-19 illness correlates with the risk of developing cardiac issues in the post-acute phase [7]. It is unclear whether prior immunity from vaccines or previous infections protects against these complications. Therefore, the increased risk of cardiovascular diseases is envisioned to be a significant health problem for many years to come, and needs to be monitored [8].

## 2 Objectives

A systematic review of the main acute cardiovascular complications secondary to COVID-19 with special emphasis on pathophysiology and mechanisms of disease. Secondly, we reviewed the effects of COVID-19 syndrome in cardiovascular health based on the relevant medical literature.

## 3 Methods

A systematic review of the literature was conducted. The studies were identified by searching electronic databases (Medline via PubMed) from 2020 to 2024. Reports were included if they had the words: COVID-19 and/or cardiovascular disease. A secondary search included the terms: long COVID-19 and cardiovascular disease. Reports which discussed any association between COVID-19, and cardiovascular effects were included, those which discussed other associations were excluded. Literature reviews into COVID-19 and Long COVID were used to confirm that studies were not missed by our original search criteria. Prospective and retrospective cohort studies were included, as well as cross-sectional studies assessing COVID-19, the heart and vasculature.

To distinguish the effects of acute COVID-19 vs Long COVID, we used a cut off of 2 months. Studies investigating effects earlier than 2 months post infection were considered acute effects while those investigating effects beyond 2 months post infection were considered Long COVID.

The search was limited to original research papers published in English.

## 4 Results

### 4.1 COVID 19. Effects on the heart on the acute setting

#### 4.1.1 Pathophysiology

The pathophysiology of SARS-CoV-2 infection revolves around its interaction with the host angiotensin-converting enzyme 2 (ACE2) receptor, facilitating cellular entry. ACE2 receptors are prominently expressed in the lungs, heart, and blood vessels. Cardiovascular disease (CVD) associated with COVID-19 is likely a consequence of the dysregulation of the ACE/ACE2 system, first due to SARS-CoV-2 infection but exacerbated by comorbidities such as hypertension [9], [10], [11]. Additionally, acute lung injury after COVID-19 increases cardiac workload, which is particularly relevant in heart failure (HF) [10], [12].

Other molecules, like neuropilin-1, have the potential to facilitate SARS-CoV-2 cell entry and infectivity, though their significance in the context of CVD remains unclear. A cytokine storm, driven by an imbalance of T-cell activation and dysregulated release of interleukin (IL)-6, IL-17, and other cytokines, may contribute to CVD in COVID-19. Immune system activation, coupled with alterations in immunometabolism, may lead to plaque instability and contribute to acute coronary events [9].

Cardiac effects of COVID-19′s are heterogeneous, with multiple concurrent histopathological findings observed in autopsies of non-survivors. These findings commonly include microthrombi and cardiomyocyte necrosis. Macrophages often infiltrate the heart, although they may not fulfill histologic criteria for myocarditis. The high prevalence of microthrombi and inflammatory infiltrates in fatal COVID-19 cases raises concerns about subclinical cardiac pathology in recovered patients. Molecular studies suggest that SARS-CoV-2 infection of cardiac pericytes, dysregulated immunothrombosis, and pro-inflammatory and anti-fibrinolytic responses underlie COVID-19 cardiac pathology [13]. The impact of mild COVID-19 on the heart remains unknown [12].

Hyperactivated platelets, some containing viral RNA, secrete factors that activate microvascular endothelial cells and weaken endothelial junctions, promoting immunothrombosis. The temporal and causative relationships of these events are complex and have not been fully defined yet.

A notable feature of acute COVID-19 is endothelial dysfunction in the coronary microvasculature, not affecting larger epicardial coronary vessels. This dysfunction includes pro-inflammatory endothelial cell activation, loss of junctional integrity, and cell death. SARS-CoV-2 has been found to directly infect human cardiomyocytes, including those derived from induced pluripotent stem cells, in a manner dependent on ACE2 and cathepsin [10]. Recent evidence suggests that pericytes, rather than endothelial cells, may serve as the primary site of SARS-CoV-2 infection in the heart, causing endothelial damage. In vitro studies indicate that exposure to the SARS-CoV-2 spike protein can alter pericyte function, impacting endothelial cell support and promoting inflammation and endothelial cell death, which might underlie acute myocarditis [12], [14].

To fully understand the pathophysiology of cardiac damage in SARS-CoV2 infection, further investigation including larger sample sizes, clinical stratification, standardized analytic frameworks, and ample tissue samples are necessary.

### 4.2 Biomarkers

Cardiomyocyte injury, measured by cardiac troponin T/I levels, and hemodynamic stress, assessed through BNP and N-terminal B-type natriuretic peptide (NTproBNP) levels, can occur in COVID-19, similar to other pneumonia cases. The extent of these biomarkers' elevation is linked to the severity of the disease and its mortality rate [10]. Slight increases in cardiac troponin T/I and/or BNP/NT-proBNP levels are typically a result of pre-existing heart conditions or acute injury/stress caused by the virus. If there are no typical angina chest pain or ischemic ECG changes, patients with mild elevations (e.g., <2–3 times the upper limit of normal) do not require further investigation or treatment for type 1 myocardial infarction (T1MI) [10]. Measuring cardiac troponin T/I levels is recommended if there is clinical suspicion of T1MI or new onset left ventricular dysfunction. Regardless of diagnosis, monitoring cardiac troponin T/I may aid in prognosis and risk assessment [10]. D-dimer levels reflect activated coagulation, a significant aspect of COVID-19. Given the involvement of endothelial inflammation and venous thromboembolism (VTE) in COVID-19, serial D-dimer measurements could assist in determining which patients should undergo VTE imaging and possibly receive higher than prophylactic anticoagulation doses [10].

### 4.3 Myocardial infarction

The waves of severe COVID-19 patients disrupted medical services and patient arrival times at emergency departments for those suspected of having acute coronary syndrome. This disruption led to an increase in door-to-balloon time, scar size (as confirmed through cardiac magnetic resonance imaging), and the incidence of mechanical complications [10], [15]. In addition, the COVID-19 pandemic has caused delays in the time from initial medical contact to revascularization for patients with ST-segment elevation myocardial infarction (STEMI) and out-of-hospital cardiac arrest. In some studies, SARS-CoV-2 infection has even been classified as a risk factor or trigger for MI [16] with STEMI rates as high as 2.5 % [16]. Factors affecting mortality include age, type of MI (STEMI or NSTEMI), diabetes mellitus (DM), respiratory failure, and ejection fraction (EF) [16]. Mortality rates appear to differ between various pandemic waves, with some studies showing increasing trends and others decreasing [16].

However, despite these associations, studies examining the global impact of the COVID-19 pandemic on the mortality of MI patients have produced inconsistent results. Some studies have reported higher in-hospital mortality, while others have not [15]. To summarize, there is a complex interplay between COVID-19 and acute myocardial infarction, with elevated mortality risk for patients dealing with both conditions. We highlight the importance of addressing respiratory failure and cardiac shock in these patients. Extended observation beyond in-hospital care is needed to fully understand the impact of COVID-19 on patient outcomes.

### 4.4 Heart failure

The coronavirus-2019 disease (SARS-CoV-2) is associated with elevated morbidity and mortality [17], [18]. Since the onset of the pandemic, cardiovascular disease has been identified as a risk factor for COVID-19, with cardiovascular complications being common over the course of acute disease as well as the following months and years [19].

Heart failure (HF) represents 3 % of hospital admissions and is the number one cause of hospitalization in patients over the age of 65 [20]. The aging population and healthcare advancements are the main factors associated with this growing increase in admissions due to HF [21].

To date, few studies have been published regarding mortality in patients with HF and COVID-19 infection. Álvarez-García *et al.* found that patients with a history of HF show a significant increase in mortality and need for invasive mechanical ventilation associated with COVID-19 infection, regardless of their ventricular ejection fraction [22]. Rey *et al.* concluded that patients infected with COVID-19 are at higher risk of developing HF during hospital admission, with high mortality rates [23].

Heart failure represents one of the main reasons for hospitalization in our environment [24], [25]. In recent years, the mortality rate for HF has been trending downwards thanks to the incorporation of different treatments that have shown significant benefits [26], the high level of adherence to guidelines by health professionals, and the change of components of disease decompensation such as ischemic heart disease, which has also been decreasing in recent years [27].

In a recent study, Bromage *et al.* showed a clear increase in the mortality of patients admitted for HF and COVID-19, though it is unknown whether this is due to true mortality or due to a selection of critical patients who were admitted during the pandemic [28].

It has shown that the presence of HF, both in patients with a prior history and those who developed it as a complication during admission, is associated with high hospital mortality rates close to 50 %. In addition, it makes clear that patients who experienced decompensation due to acute HF while admitted died at a higher rate than patients with a history of HF prior to admission, with the former affecting mortality more than the latter. This reflects the impact of COVID-19 on developing acute HF. These percentages represent a significant increase compared to the mortality described in previous studies of patients hospitalized with HF, which range from 9.5 % to 11 % [29], [30].

The most frequent causes of death in patients with a history of HF have been described as decompensated HF, sudden death, and non-cardiovascular causes [31], [32]. Currently, SARS-CoV-2 is the primary cause of death in hospitalized HF patients.

Older age, elevated comorbidity burden, and degree of dependency are characteristic elements of patients with HF hospitalized due to COVID-19. A Spanish study showed this clinical profile, with figures close to 80 % for arterial hypertension, 40 % for atrial fibrillation, 20 % for diabetes mellitus, 17 % for moderate-severe chronic kidney disease, 25 % for obesity, and 38 % for severe functional dependency [33]. It represents a continuation of previous studies which have reported similar figures for these HF-related pathologies [33], [34].

Old age and chronic conditions are just some of the various clinical conditioning factors that could favor its development and predispose patients to the onset of complications and adverse events [10], [35]. Old age has been determined to be one of the main factors associated with higher mortality due to COVID-19 [10].

On the other hand, the importance of assessing clinical condition at the time of admission must be mentioned. Patients who present in critical condition will have a significantly worse prognosis tan those presenting without these characteristics, with the former requiring stricter monitoring and follow-up. Other studies have also stressed this issue and its severity in COVID-19 illness [16].

In addition, the presence of certain altered biological markers in patients with HF can be aggravated by COVID-19 infection. In this way, kidney failure and high CRP and LDH levels are significantly associated with an increase in mortality. However, other markers of poor prognosis in COVID-19, such as lymphopenia or elevated D-dimer levels [15], are not related to higher mortality in patients with HF.

In any case, though the variables of heart rate and elevated LDH and CRP have been statistically associated with mortality, we must bear in mind that the association of these factors has been less important from the clinical perspective than other variables such as dependency or age. All of these factors, which are easily identifiable when patients are being admitted to hospital, define a patient profile that requires special care due to the high risk of complications and death that they entail. It is also worth noting that we have taken into account the characterization of HF such as etiology, the New York Heart Association functional class, the left ventricular ejection fraction, the level of natriuretic peptides, and the proportion of patients receiving other treatments such as beta blockers, neprilysin receptor antagonists, aldosterone antagonists, or diuretics.

### 4.5 Takotsubo Cardiomyopathy

Takotsubo Cardiomyopathy (TTC) is a cardiac condition that typically arises from emotional distress but, in an intriguing twist, has been identified in some COVID-19 patients, associated with physical stress [17]. The clinical presentation of COVID-19 patients with TTC included symptoms such as shortness of breath and chest pain. Electrocardiograms revealed a range of abnormalities, from ST-segment elevations to atrial fibrillation, T-wave inversion, and sinus tachycardia [18].

Remarkably, the biomarker levels in COVID-19 patients with TTC were notably different from COVID-19 patients with non-TTC myocardial injuries or patients without myocardial injury. TTC patients displayed higher cardiac biomarkers like cardiac troponin I and creatine kinase myocardial band, while non-TTC myocardial injury patients had elevated inflammatory and prothrombotic biomarkers such as interleukin-6, ferritin, and d-dimer. They also exhibited left ventricular dysfunction, with a lower median left ventricular ejection fraction (LV EF) when compared to patients with other types of myocardial injuries and those without myocardial injury [20], [36].

TTC should be considered as a possible diagnosis in COVID-19 patients with myocardial injury, where transthoracic echocardiogram becomes very valuable, guiding management and deciding on the necessity for further invasive work-up. The development of TTC in these patients might be due to catecholamine-induced microvascular dysfunction, triggered by the metabolic, inflammatory, and emotional distress associated with COVID-19 [21].

### 4.6 Myocardial injury and myocarditis

Evidence of acute cardiac injury, as indicated by elevated troponin levels, is a common occurrence among patients suffering from COVID-19. The precise mechanisms underlying myocardial injury in COVID-19 are still shrouded in uncertainty, though several factors have been proposed, including direct viral effects, hypoxia, and immune-mediated processes. While myocarditis has been postulated as a potential complication, it seems to be a relatively infrequent outcome of SARS-CoV-2 infection.

One valuable tool for assessing myocarditis in such cases is Cardiac Magnetic Resonance (CMR), which offers advantages in evaluating cardiac structure and function in patients with suspected myocarditis, pericarditis, or myopericarditis [37]. It is noteworthy that even when these criteria are not met, abnormal CMR findings can still carry clinical significance. Coupling Cardiopulmonary Exercise Testing (CPET) with CMR has revealed that lower maximal oxygen consumption (VO2max) is associated with initial CMR abnormalities in patients who have recovered from COVID-19.

Moreover, it is crucial to recognize the transient nature of myocardial inflammation in COVID-19. Fluoro-deoxy-glucose (FDG) Positron Emission Tomography (PET) proves to be more sensitive than CMR in detecting myocardial inflammation and is highly specific in diagnosing chronic myocarditis [38]. In COVID-19 patients who test positive for FDG-PET, there are often indications of ventricular dysfunction and elevated systemic inflammatory biomarkers. However, on subsequent follow-up, these abnormalities tend to resolve, underscoring the temporary nature of myocardial inflammation in this context [38].

Furthermore, the presence of myocardial injury appears to be predictive of in-hospital mortality in patients afflicted with severe COVID-19. Those with cardiac involvement demonstrate a higher mortality rate [21]. While some studies have identified cardiac abnormalities through various imaging and diagnostic tools, the exact mechanisms driving myocardial injury in the context of COVID-19 remain a subject of ongoing research. There is growing evidence to suggest that systemic inflammation and hypoxia may play pivotal roles, with viral involvement possibly leading to viral myocarditis in certain cases.

### 4.7 Arrhythmias

Recent articles highlight the growing concern of atrial fibrillation (AF) and ventricular arrhythmias in COVID-19 patients, particularly among those aged 60 or older and those with severe COVID-19. Studies indicate that AF, including new-onset cases, independently associates with a heightened risk of all-cause mortality in hospitalized COVID-19 patients [39]. Comparing survivors and non-survivors, survivors had statistically significant lower initial heart rates compared to non-survivors [40]. Sinus tachycardia was the most common arrhythmia among monitored COVID-19 patients, occurring more frequently in non-survivors. Premature ventricular complexes and non-sustained ventricular tachycardia were also observed but with no significant difference in incidence between survivors and non-survivors. Sustained ventricular tachycardia and ventricular fibrillation were rare occurrences. These findings are not consistent in all series [41].

**Fig. 1:**
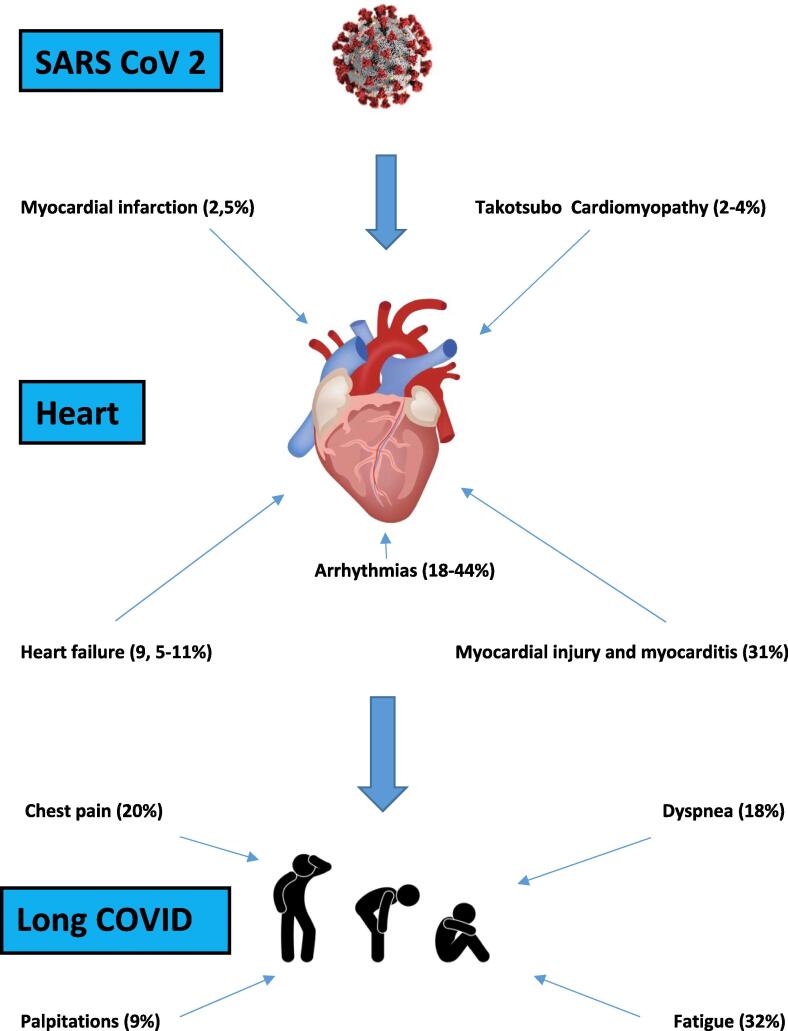
Cardiovascular complications of COVID-19: acute setting and post-acute sequela (rates).

Fig. 1 shows the main cardiovascular complications of COVID-19.

## 5 Long COVID and cardiovascular health

### 5.1 Long COVID

Post-acute sequelae of SARS-CoV-2 infection (PASC), also known as Long COVID, is the continuation or emergence of symptoms at least 12 weeks after COVID-19. Over 200 symptoms have been associated with PASC [10], [12], which are likely caused by several different underlying mechanisms [8] covering multiple organ systems but including many cardiovascular complaints [15], [16]. Various studies have raised concerns about the impact of long COVID on the cardiovascular system, including increased risk of new onset hypertension and de novo heart failure [17]. One such example is increased troponin levels during acute infection suggested poorer patient outcomes 12 months later, including increased overall mortality and increased cardiac complications [18].

As stated above, COVID-19 can cause significant cardiac damage, which is unlikely to resolve for weeks after infection. The most common persistent symptoms associated with cardiovascular abnormalities are chest pain and palpitations, being reported in around 20 % and 9 % of COVID-19 survivors at 60 days post infection, respectively [20], [36].

Cardiovascular magnetic resonance imaging (CMR) found that 78 % of patients had cardiac abnormalities at 71 days post COVID-19 diagnosis [21]. These included myocardial inflammation, myocardial injury, and pericardial inflammation. Comparing hospitalised and non-hospitalised patients revealed that the severity of the abnormalities was not related to the severity of the initial COVID-19 infection, suggesting that even mild cases of COVID-19 can lead to cardiac damage [21]. Other studies have used similar techniques but found lower rates of cardiac abnormalities, such as myocardial scarring which was identified in some asymptomatic or mild cases (4 % with LGE myocardial scarring) [39] including athletes (2.3 % and remain asymptomatic) [41] 22 days post diagnosis. Hospitalized patients with long COVID still showed cerebrovascular impairment 6 months after discharge [42]. Short term studies therefore suggest that COVID-19 causes a number of pathologies to the cardiovascular system, but the Long term effects of these infections is much more important.

Longer term, the risk of cardiac complications is still notable. Chest pain and palpitations were reported in 5 % and 9 % of survivors respectively at 6 months [20]. The long-term consequences of cardiac damage are not yet known, but it is possible that patients who survive COVID-19 may be at increased risk of heart failure, arrhythmias, myocardial inflammation/ injury and other cardiac complications. Electronic health records from the US Veterans Health Administration (VHA) found markedly higher hazard ratios in patients who survived COVID-19 for a range of cardiovascular outcomes, most notably myocarditis [43]. This cohort of over 5 million people are older, more male and have a range of unfavorable risk factors such as being more likely to smoke. Xie et al’s data therefore most likely provides us with an upper boundary for increased CVD risk post COVID-19, as other studies on different cohorts have found a non-clinically significant change one year post infection [44]. Electronic health data from the UK showed doubling of the rate of major adverse cardiac events (MACE) occurring more often in hospitalized patients post-COVID than in exactly matched controls [45].

Regardless of the mechanism, the cardiac effects of long COVID are a cause for concern. Recent studies have documented cardiac abnormalities in patients with long COVID, such as myocardial inflammation, myocardial injury, and pericardial inflammation. Care strategies of people who survived the acute episode of COVID-19 should include attention to cardiovascular health and disease for at least the first 12 months after the acute infection. Some studies are already suggesting anticoagulant treatment in hospitalized patients with imaging-confirmed venous thromboembolism (VTE) for at least 3 months [37], [46], [47]. Further research is needed to better understand the mechanisms underlying the cardiac effects of long COVID and to develop effective strategies for prevention and treatment.

Based on the above data, recent COVID-19 may be seen as a risk factor in patients for myocarditis and heart dysfunction. Whether this risk decreases over time is important for fully understanding how to factor COVID-19 into risk analysis along with factors such as smoking, high cholesterol and obesity [48]. The medical community also needs to understand whether the elevated risk of cardiac disease eventually decreases to baseline levels after COVID-19, whether reinfections increase cardiac risk as much as the primary infection, and whether vaccinations have a protective effect. Circulating SARS-CoV2 may otherwise cause a permanent increase in global cardiovascular disease.

### 5.2 Vaccinations and long-term cardiac risk after COVID-19

Vaccinations have been highly successful in reducing the symptoms and risk of death caused by COVID-19 [49], but questions remain about whether this extends to increased cardiovascular risks as discussed above. However, after initial controversy around the risk of myocarditis after COVID-19 vaccination, the risk of vaccine-induced cardiac complications remains rare [50], [51] and vaccines appear to be cardioprotective overall [52].

Given that vaccines reduce many COVID-19 symptoms, as well as the severity of at least some Long COVID symptoms [53], [54], [55], the possibility that vaccines have reduced cardiovascular risk is an important consideration. Evidence from the US suggests that vaccines have reduced the risk of MACE [56], with similar supporting data from Electronic Health Records in the UK [57], Israel [58] and Korea [59].

The data therefore suggest that the population-level risk of adverse cardiovascular events is likely to be lower now than it was in 2020–21 when most of the global population was unvaccinated. Reinfections with newer SARS-CoV-2 variants are likely to increase cardiovascular disease risk [60], although the effect may be more marginal in a population with strong immunity. Whether further vaccinations in the future are required to keep the risk down is not currently clear, and population-level monitoring of cardiovascular events after COVID-19 should continue [53].

### 5.3 Microclotting as an association factor of Long COVID

Long COVID is characterized by a constellation of symptoms affecting many organs, beyond the cardiovascular system. Notably, some have proposed that microclotting – fibrinogen deposits which block small blood vessels – may be the cause of non-cardiovascular Long COVID symptoms [61]. These microclots are proposed to disrupt blood flow in the microvasculature, and could cause fatigue, myalgia, dyspnea, brain fog, and other symptoms associated with Long COVID.

Supporting this hypothesis, microclots have been observed in the vasculature of patients with Long COVID, at least 2 months after diagnosis [62], [63]. These microclots could be induced by acute SARS-COV-2 infection as the SARS-CoV-2 spike protein S1 subunit can induce agglutination of fibrinogen and platelets, when added to healthy samples [61] and that monocytes express pro-thrombotic genes after SARS-CoV-2 infection [40]. The same researchers later used mass spectrometry analysis to reveal that these microclots have entrapped inflammatory mediators such as α(2)-antiplasmin (α2AP), von Willebrand factor (VWF), and Serum Amyloid A (SAA) [64], which they propose might mask the measurement of these factors in patient’s blood. These microclots have deposits of fibrinogen in an amyloid structure, making them resistant to fibrinolysis, which might allow them to persist for very long times in the blood [61], [65].

This raises several questions, such as why only some patients go on to develop Long COVID, if microclots/prothombotic states are prevalent in most acute COVID illness phases and why/how vaccination might help to alleviate this. Additionally, as many people report spontaneous recovery from Long COVID symptoms over time, it would be good to see longitudinal decline in microclots as patients recover. Whether patients treated with anticoagulants experience fewer Long COVID symptoms would be interesting to see, however the universal use of therapeutic dose anticoagulation in COVID-19 infection was discontinued as more data showed that risk of bleeding outweighed generic use of anticoagulation.

### 5.4 Treatments for Long COVID focusing on cardiovascular complications

Based on the hypothesis that microclots are a cause of Long COVID, anticoagulants ought to act as a treatment for Long COVID. Indeed, triple anticoagulant therapy [66] of 91 patients reporting Long COVID symptoms led the majority to report an improvement in their symptoms. Apheresis [67] is reported to help reduce Long COVID symptoms, and this could be due to removal of microclots. A small pilot study of 13 patients trialed slow diaphragmatic breathing exercises as a method to reduce heart rate variability and found improvements in various scores measuring Long COVID symptomology [68].

Potentially, prophylactic treatments during the acute phase of COVID-19 may also reduce the risk of Long COVID. One such example is nirmatrelvir, an antiviral protease inhibitor which reduces the risk of post-COVID-19 symptoms in patients by 26 % if given during the acute phase of infection [43], [69]. If microclotting plays a role in driving Long COVID symptoms, nirmatrelvir might reduce the rate of microclotting caused by initial infection. A placebo-controlled trial investigating whether nirmatrelvir reduces Long COVID symptoms is underway (NCT05668091), and the effects on specific symptoms such as cardiovascular risks will be of particular interest.

As has been discussed already, Long COVID manifests as a wide range of symptoms which likely have multiple underlying causes [8]. It is therefore likely that treatments may only work for certain symptoms or symptom clusters. Ivabradine is a pacemaker current inhibitor (NCT05481177) and efgartigimod is a neonatal Fc receptor blocker (NCT05918978) and both are being trialed to treat tachycardia or POTS in Long COVID patients.

As there are currently no treatments for Long COVID, positive results from any of these studies would signal potential benefits for patients in the future.

## 6 Conclusions

Overall, this review brings together a myriad of literature which agree that cardiovascular complications of COVID-19 are numerous and life-threatening. Complications include myocardial infarction, heart failure, Takotsubo cardiomyopathy, myocarditis and arrhythmias. An understanding of this by cardiac clinicians is important given the repercussion and possible complications caused by COVID-19. Additionally, early interventions such as the treatment of infection with remdesivir can lead to a decrease in cardiac-related mortality and morbidity associated with SARS-CoV-2.

Long COVID can manifest as defects in cardiovascular health. Although the mechanisms of Long COVID are not yet completely clarified, it is essential to understand its pathophysiology to locate new therapeutic targets. Microclotting induced by SARS-CoV-2 infection could be a factor causing Long COVID and therefore a therapeutic target. Oral anticoagulation has shown encouraging results, but more studies are necessary in this regard.

## Funding

This work was funded by an NIHR and Wellcome Trust Award – G112259 and 226389/Z/22/Z, respectively to NS and a Wellcome Trust Award – 225023/Z/22/Z to BAK.

## CRediT authorship contribution statement

**B.A. Krishna:** Writing – review & editing, Writing – original draft. **M. Metaxaki:** Writing – original draft. **N. Sithole:** Writing – review & editing. **P. Landín:** Writing – original draft. **P. Martín:** Writing – original draft. **A. Salinas-Botrán:** Writing – review & editing.

## Declaration of competing interest

The authors declare that they have no known competing financial interests or personal relationships that could have appeared to influence the work reported in this paper.
